# Clinical characteristics of lower extremity deep vein thrombosis in young vs. middle-aged adults: a single-center retrospective study

**DOI:** 10.3389/fcvm.2024.1381391

**Published:** 2024-04-26

**Authors:** Yaqiu Zhang, Haijun Wei, Yang Liu, Qiqi Wang, Chunshui He

**Affiliations:** Department of Vascular Surgery, Hospital of Chengdu University of Traditional Chinese Medicine, Chengdu, Sichuan, China

**Keywords:** lower extremity deep vein thrombosis, young, PTS, thrombus recurrence, inferior vena cava thrombosis

## Abstract

**Background:**

The incidence of deep vein thrombosis (DVT) in the lower extremities is increasing in the younger population. However, there are fewer reported comparisons in the literature for lower extremity DVT.

**Methods:**

Patients aged <40 years admitted with lower-extremity DVT between January 2018 and December 2023 were retrospectively analyzed and followed up for 1 year.

**Results:**

A total of 61 patients were included in the study and divided into two groups: 33 patients over 30 years of age (middle-aged group) and 28 patients under 30 years of age (young group). A significant gender difference was observed, with a higher proportion of males in the young group compared to the middle-aged group (*P* < 0.001). Five patients in the young group were treated with anticoagulation alone, whereas all patients in the middle-aged group underwent endovascular therapy. A higher prevalence of inferior vena cava thrombosis in the young group compared to the middle-aged group (60.71% vs. 33.3%, *P* = 0.032). The proportion of iliac vein stenosis was significantly higher in the middle-aged groups than in the young group (*P* = 0.002). There was no statistically significant difference in venous function scores (Villalta and rVCSS) between the two groups during both the preoperative period and the postoperative follow-up (*P* > 0.05). The incidence of lower-extremity DVT post-thrombotic syndrome and thrombus recurrence was higher in the young group than in the middle-aged group at 1 year postoperatively (PTS: 78.57% vs. 33.3%, *P* < 0.001, and thrombus recurrence: 28.57% vs. 9.09%, *P* < 0.05). Univariate and multivariate analyses revealed that inferior vena cava thrombosis was an independent risk factor for severe DVT post-thrombotic syndrome and recurrent DVT (*P* < 0.05), whereas gender was an independent risk factor for recurrent DVT (*P* < 0.05).

**Conclusions:**

This study suggests differences in the clinical characteristics and prognosis of lower-extremity DVT.

## Introduction

The prevalence of lower extremity deep vein thrombosis (DVT) is primarily attributed to factors that affect blood flow, such as tumors, aging, hypercoagulability and prolonged immobilization (1, 2). These factors traditionally result in a lower incidence of DVT in younger individuals. However, in recent years, the prevalence of DVT has been on the rise among the younger population in China (3). The formation of DVT can easily lead to post-thrombotic syndrome (PTS), a condition with later symptoms that significantly impact the patient's quality of life. This is particularly challenging for younger patients to accept. PTS, stemming from DVT in the lower extremities, inevitably diminishes the patient's quality of life, posing an unacceptable burden for the younger demographic. Recent literature suggests that DVT characteristics in young adults differ from those in older patients (4–6). However, detailed comparisons of DVT in China are scarce. Therefore, this study aims to conduct a retrospective analysis of 61 patients under the age of 40 admitted to our center. The analysis provides a preliminary exploration of DVT in the younger age group.

## Materials and methods

### Study design

A retrospective analysis was conducted on 61 patients under 40 years of age who were diagnosed with lower extremity deep vein thrombosis (DVT) from January 2018 to December 2023. The study was conducted at the Department of Vascular Surgery, Affiliated Hospital of Chengdu University of Traditional Chinese Medicine. Enrolled participants were confirmed to have venous thrombosis above the popliteal vein through lower extremity venous ultrasound. Only patients who were first-time diagnosed with lower extremity DVT and received anticoagulation therapy or underwent venography were included in the study. Exclusion criteria included patients who did not receive anticoagulation therapy or undergo venography.

### Treatment

All admitted patients received standard anticoagulation therapy using low molecular weight heparin sodium or rivaroxaban tablets (anticoagulant course >3 months). Additionally, all enrolled patients underwent lower extremity venography. For patients with acute iliofemoral vein thrombosis (onset within 14 days with markedly elevated D2 polymers), after communication with the patients and their families, the inferior vena cava filter is implanted and Angiojet thrombus is aspirated (7, 8). For cases with refractory thrombus, the surgeon will perform catheter contact thrombolysis (CDT) according to the situation. Balloon dilatation was performed for patients with iliac vein compression, and the decision to implant a stent was made by the surgeon as needed. Compression therapy was applied at the end of the procedure to alleviate lower limb swelling.

### Variables evaluated

In addition to basic information such as age, gender, and weight, the study collected indicators related to the increased risk of venous thrombosis, tumors, marital history, history of prolonged braking, and various biochemical indexes (hemoglobin, leukocytes, neutrophil counts, D2 polymorphisms, protein C, protein S, etc.). The location of lower extremity DVT was recorded by imaging, and the intraoperative Angiojet documented the thrombus location. The use of Angiojet thrombus aspiration catheter, CDT, balloon, and thrombus clearance were also indicated.

### Outcome

All patients underwent follow-up at the hospital and were contacted by telephone at 6 and 12 months postoperatively. Follow-up included lower extremity venous ultrasound, Villalta score assessment, and Revised venous clinical severity scores (rVCSS). A Villalta score exceeding 15 indicated severe lower extremity post-DVT syndrome.

### Statistical analysis

Normality of the quantitative data was assessed, and descriptive statistics were applied accordingly. To compare the young and middle-aged groups, *t*-tests were used for normally distributed data, Wilcoxon rank sum tests for non-normally distributed data, and chi-square tests/exact tests for categorical data. Risk factors for post-thrombotic syndrome (PTS) and thrombosis recurrence were analyzed using univariate and multivariate logistic regression. Statistical significance was determined at a significance level of *P* < 0.05. All analyses were performed using SAS statistical software (version 9.4, Cary, NC).

## Results

### Population characteristics, lesion characteristics, and surgical characteristics

This study included patients diagnosed with lower extremity deep vein thrombosis (DVT), 33 of whom were over 30 years old and 28 under 30. The proportion of males with lower extremity DVT was significantly higher in the younger population compared to the middle-aged and young adult population (78.57% vs. 33.3%, *P* < 0.001). The analysis of listed risk factors (Table 1) showed no history of tumors in either group. Family history was observed in only one case in each group. The young group had more case of recent hormone use, trauma, and history of deep vein thrombosis compared to the middle-aged group. On the other hand, the young group had fewer cases of history of braking, infections, and surgeries than in the middle-aged group. Table 2 show thathemoglobin levels were slightly higher in the young group (4.29 ± 0.67 vs. 4.35 ± 0.53, *P* = 0.705) among the listed blood indices, Fibrinogen was elevated in the middle-aged group (4.19 ± 1.36 vs. 4.84 ± 2.62, *P* = 0.244). Protein C deficiency was found in two cases, and protein S deficiency was found in one case in the young group, while none were found in the middle-aged group. As shown in Table 3, the analysis of the lesions showed a significantly higher incidence of inferior vena cava thrombosis among individuals under 30 years of age. Additionally, this group had a lower rate of iliac vein stenosis in this group (57.14% vs. 90.19%, *P* = 0.002).

**Table 1:** Patient demographics.

Variable	Total (*n* = 61)	Groupe		Statistic	*P*
		<30 year (*n* = 28)	>30 year (*n* = 33)		
Age, mean ± SD	29.92 ± 7.01	23.36 ± 3.98	35.48 ± 3.04	−13.468	<.001
BMI, mean ± SD	23.37 ± 2.96	22.96 ± 3.71	23.71 ± 2.13	0.951	0.347
Gender, *n* (%)				12.484	<.001
F	28 (45.9)	6 (21.43)	22 (66.67)		
M	33 (54.1)	22 (78.57)	11 (33.33)		
Hypertension, *n* (%)	1 (1.64)	0 (0.00)	1 (3.03)	–	1.000
Diabetes, *n* (%)	0	0	0	–	–
Risk factors present % (*n*)					
Cancer	0	0	0	–	–
Marital history, *n* (%)	13 (21.31)	3 (10.71)	10 (30.30)	3.466	0.063
Family history, *n* (%)	2 (3.28)	1 (3.57)	1 (3.03)	–	1.000
Hormonal therapy, *n* (%)	4 (6.56)	3 (10.71)	1 (3.03)	0.475	0.491
Recent surgery (2 months), *n* (%)	25 (40.98)	9 (32.14)	16 (48.48)	1.673	0.196
Recent trauma (2 months), *n* (%)	7 (11.48)	4 (14.29)	3 (9.09)	0.053	0.817
Immobilization, *n* (%)	20 (32.79)	6 (21.43)	14 (42.42)	3.030	0.082
History of infection, *n* (%)	10 (16.39)	2 (7.14)	8 (24.24)	2.104	0.147
May Thurner syndrome, *n* (%)	1 (1.64)	1 (3.57)	0 (0.00)	–	0.459
DVT staging, *n* (%)				–	0.459
Acute	60 (98.36)	27 (96.43)	33 (100.00)		
Chornic	1 (1.64)	1 (3.57)	0 (0)		

**Table 2:** Hematology indicators.

Variable	Total (*n* = 61)	Groupe		Statistic	*P*
		<30 year (*n* = 28)	>30 year (*n* = 33)		
White blood cell, 10^9^ L, Mean ± SD	8.25 ± 1.93	8.59 ± 1.95	7.96 ± 1.90	1.278	0.206
Erythrocyte, 10^12^ L, mean ± SD	4.32 ± 0.59	4.29 ± 0.67	4.35 ± 0.53	−0.381	0.705
Hemoglobin, g/L, mean ± SD	134.23 ± 18.55	138.82 ± 19.03	130.33 ± 17.48	1.815	0.075
Fibrinogen, g/L, Mean ± SD	4.54 ± 2.14	4.19 ± 1.36	4.84 ± 2.62	−1.177	0.244
D2 polymersug/ml, mean ± SD	6.77 ± 5.66	6.90 ± 5.78	6.66 ± 5.66	0.165	0.869
Protein C deficiency, *n* (%)	2 (3.28)	2 (7.14)	0 (0.00)	–	0.207
Protein S deficiency, *n* (%)	1 (1.64)	1 (3.57)	0 (0.00)	–	0.459

**Table 3:** Summary of treatment characteristics.

Variable	Total (*n* = 61)	Group		Statistic	*P*
		<30 year (*n* = 28)	>30 year (*n* = 33)		
Classification, *n* (%)					
Central	21 (34.43)	9 (32.14)	12 (36.36)	0.120	0.730
Mixed	37 (60.66)	18 (64.29)	19 (57.58)	0.286	0.593
Inferior vena cava thrombosis, *n* (%)	28 (45.9)	17 (60.71)	11 (33.33)	4.573	0.032
Anticoagulant therapy alone, *n* (%)	5 (8.2)	5 (17.86)	0 (0.00)	4.265	0.039
Angiojet, *n* (%)	37 (60.66)	17 (60.71)	20 (60.61)	0.000	0.993
Catheter directed thrombolysis, *n* (%)	23 (37.7)	8 (28.57)	15 (45.45)	1.838	0.175
Balloon, *n* (%)	34 (55.74)	15 (53.57)	19 (57.58)	0.098	0.754
Stent, *n*, (%)	20 (32.79)	9 (32.14)	11 (33.33)	0.009	0.922
Iliac vein stenosis or occlusion, *n* (%)	46 (75.41)	16 (57.14)	30 (90.91)	9.314	0.002
Thrombus resolution, *n* (%)	31 (50.82)	16 (57.14)	15 (45.45)	0.828	0.363
Elastic stockings, *n* (%)	61 (100)	28 (100)	33 (100)	–	–

### Follow-up results

**Figure 1:**
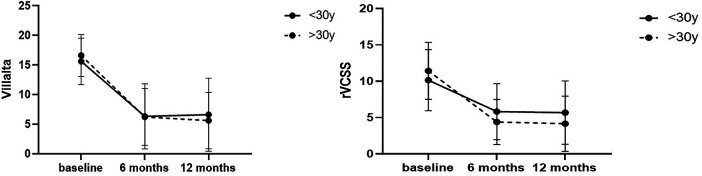
The score of vilata and revised venous clinical severity scores (rVCSS).

The mean Villalta scores improved at the 6-month and 12-month follow-ups for the young (baseline, 15.60; 6 months, 6.32; 12 months, 6.60; *P* < .0001), and middle age (baseline, 16.60; 6 months, 6.21; 12 months, 5.60; *P* < .0001) groups (Figure 1A). No difference in 6-month and 12-month Villalta score improvement was observed between the groups (*P* > 0.05). The mean rVCSS at baseline, 6 months, and 12 months was 10.14, 4.39, and 5.67, respectively, (6 months, *P* < 0.001; 12 months, *P* < 0.001; each follow up compared with baseline) for patients in the young group and 11.42, 5.82, and 0.15, respectively, (*P* < .0001 for each follow-up) for patients in the middle-aged group. Figure 1 illustrate variation in Villalta and rVCSS distribution between the groups. Improvement in 6-month and 12-month rVCSS was not significantly different between the groups. The young group had higher scores. A Villalta score exceeding 15 points indicatS severe PTS. The incidence of PTS was significantly higher in the young group than in the middle-aged group (78.57% vs. 33.3%, *P* < 0.001). Furthermore, the recurrence rate of lower extremity DVT was significantly higher in the youth group than in the middle-aged group (28.57% vs. 9.09%, *P* < 0.05, Table 4). The study conducted univariate and multivariate logistic regression analyses to determine the relationship between PTS and recurrence of lower extremity DVT. The results showed that inferior vena cava thrombosis was an independent risk factor for both PTS and recurrence of lower extremity DVT (Tables 5, 6). Additionally, Males were identified as an independent risk factor for the recurrence of lower extremity DVT (Table 6).

**Table 4:** Follow-up results of PTS and thrombosis recurrence after 12 months.

Variable	Total (*n* = 61)	Groupe		Statistic	*P*
		<30 year (*n* = 28)	>30 year (*n* = 33)		
Severe PTS, *n* (%)	12 (19.67)	8 (28.57)	4 (12.12)	2.594	0.107
Thrombosis recurrence, *n* (%)	11 (18.03)	8 (28.57)	3 (9.09)	3.889	0.049

**Table 5:** Univariate and multifactor logistic regression analysis of clinically relevant risk factors for PTS.

Variables	Beta	S.E	*Z*	OR (95% CI)	*P*	aBeta	aS.E	aZ	aOR (95% CI)	aP
Age	−0.06	0.05	−1.19	0.95 (0.86–1.04)	0.235	−0.03	0.05	−0.55	0.97 (0.88–1.07)	0.580
Male	1.14	0.73	1.57	3.12 (0.75–12.95)	0.116	1.14	0.73	1.57	3.12 (0.75–12.95)	0.116
Anticoagulant therapy alone	0.02	1.17	0.02	1.02 (0.10–10.08)	0.985	−1.42	1.39	−1.03	0.24 (0.02–3.66)	0.305
Iliac vein stenosis or occlusion	−0.55	0.70	−0.78	0.58 (0.15–2.29)	0.436	−0.68	0.90	−0.76	0.51 (0.09–2.95)	0.450
Inferior vena cava thrombosis	1.56	0.73	2.14	4.74 (1.14–19.74)	0.033	1.65	0.77	2.15	5.22 (1.16–23.56)	0.032

**Table 6:** Univariate and multifactor logistic regression analysis of clinically relevant risk factors for venous thrombosis recurrence.

Variables	Beta	S.E		OR (95% CI)	P	aBeta	aS.E	aZ	aOR (95% CI)	aP
Age	−0.07	0.05	−1.46	0.93 (0.85–1.02)	0.145	−0.04	0.06	−0.63	0.97 (0.87–1.08)	0.526
Male	2.46	1.09	2.27	11.74 (1.40–98.74)	0.023	2.46	1.09	2.27	11.74 (1.40–98.74)	0.023
Inferior vena cava thrombosis	2.88	1.09	2.64	17.78 (2.10–150.38)	0.008	3.11	1.15	2.71	22.34 (2.35–211.99)	0.007
Iliac vein stenosis or occlusion	−0.71	0.71	−0.99	0.49 (0.12–2.00)	0.323	−1.10	1.03	−1.07	0.33 (0.04–2.52)	0.287
Anticoagulant therapy alone	0.14	1.17	0.12	1.15 (0.12–11.42)	0.905	−1.99	1.46	−1.36	0.14 (0.01–2.40)	0.174

## Discussion

The literature suggests that younger patients with lower-extremity DVT are not common in clinical practice. However, in recent years, the incidence of patients with lower-extremity DVT has increased compared with the past, and although the management of lower extremity DVT has gained a lot of diagnostic and therapeutic experience in the clinic, there is still a lack of more evidence-based medical evidence regarding the clinical characteristics of younger patients with DVTas well as their postoperative conditions. In this study, a preliminary attempt was made to gain a better understanding of the younger DVT patient population by retrospectively comparing the differences in lower extremity DVT between young and middle-aged patients.

The pathogenesis and recurrence of the first episode of DVT are multifactorial, depending on the severity and number of genetic and indirect factors (9). Venous thrombosis is caused by hypercoagulable state, venous stasis, and endothelial damage. The main conditions associated with these three pathological processes are tumors, trauma, immobility, and childbirth younger patients are less likely to develop DVT due to these underlying factors. Kuo DJ's reports that- prolonged repetitive squatting position required by- baseball catchers may compress the blood vessels in the pelvic, inguinal, and popliteal regions, leading to the development of lower extremity DVT in younger baseball patients (5). In the Raymundo SRO report a patient with inferior vena cava malformation was found to have developed lower extremity DVT (10). Additionally, genetic factors such as abnormalities in blood proteins C and S and coagulation factor V can contribute to the development of lower extremity DVT. The study found elevated levels of protein C and S in patients under 30 years old, with less variation in other factors such as tumors. In the present study, it was found that males were more common among patients with DVT under 30 years of age, while females were more common among patients with DVT over 30 years of age. This difference was statistically significant.

The occurrence of post-thrombotic syndrome (PTS) and recurrent deep vein thrombosis (DVT) in young patients can have disastrous consequences. Long-term chronic venous hypertension can cause significant discomfort, and localized itching and ulceration of the lower extremities can affect the quality of life of patients. Therefore, in addition to preventing fatal pulmonary embolism in young patients with DVT, it is crucial to prevent recurrence of DVT and PTS in the lower extremities. According to the literature, severe post-thrombotic syndrome (PTS) occurs in 5%–10% of cases (11). However, this study found a significantly higher incidence of severe PTS in young and middle-aged patients. Additionally, patients under 30 years old with DVT had a significantly higher incidence of recurrent DVT and severe PTS within 1 year compared to young and middle-aged patients (Table 4). These findings warrant attention.

In 2017, an article published in the New England Journal of Medicine found no significant difference between anticoagulation alone and surgery in the management of lower extremity DVT with regards to the development of PTS in the later stages of the disease (12). However, the study's methodology suggests that different treatment modalities may impact the prognosis of lower extremity DVT (13–15). The discrepancy may be due to the previous study's prospective design, which introduced confounding factors, such as being conducted in the early stages of the disease. To ensure a meaningful comparison, it may be necessary to conduct a randomized controlled clinical trial, as there are several confounding factors to consider, such as the surgical procedure and type of thrombus. However, the study found no statistically significant difference in the rates of post-thrombotic syndrome (PTS) and lower extremity deep vein thrombosis (DVT) between the two treatment groups. It should be noted that the study had a small sample size and included age-specific patients, which limits the ability to draw conclusions about the effect of different treatment modalities on the occurrence of PTS and recurrence of thrombosis. Statin use has been shown (15) to reduce the incidence of PTS, and statins were not included in this study because the population consisted of young and middle-aged patients who were less likely to use lipid-lowering drugs.

Lower extremity DVT complicated by inferior vena cava thrombosis is rare and has a high mortality rate. Patients with untreated inferior vena cava thrombosis have a 90% risk of developing post-thrombotic syndrome (PTS), and up to 15% may develop severe PTS with chronic leg ulcers (16). Similarly, the incidence of inferior vena cava thrombosis was found to be as high as 60% in the young group in this study, with a significantly higher rate of PTS and recurrent thrombosis later in life than in the middle-aged group. Correlation analysis showed that the presence of inferior vena cava thrombosis in patients with lower-extremity DVT was an independent risk factor for PTS and recurrent venous thrombosis.

Regarding DVT recurrence, a prospective study by Dr. Legese Chelkeba et al. in an Ethiopian population showed that a history of previous DVT was an independent factor for DVT recurrence (2). In contrast, the patients enrolled in this study were all first diagnosed with thrombosis, which makes the two studies not comparable considering the age and ethnicity of the cohort in this study population. However, we found that male sex was an independent risk factor for recurrence in patients with lower-extremity DVT. This has been less reported in previous literature and further studies are needed to confirm this.

However, this study is a small retrospective analysis of rejuvenated lower extremity DVT patients, comparing those over 30 years old with those under 30 years old. While there are differences between the two age groups, the age difference is not significant enough to highlight the characteristics of rejuvenated DVT in certain factors. Therefore, multicenter prospective studies or randomized clinical controlled trials are needed to investigate the rejuvenation of deep vein thrombosis (DVT) in the lower extremities. This will increase awareness of young DVT and provide a more evidence-based approach to treatment at a later stage.

## Conclusion

Our study examines the unique characteristics of deep vein thrombosis (DVT) in young adults vs. middle-aged individuals. We found that young adults have higher rates of post-thrombotic syndrome (PTS) and recurrent DVT. We also identified inferior vena cava thrombosis as a potential independent risk factor for both PTS and DVT. Furthermore, gender was found to be a significant risk factor for recurrent thrombosis. These findings emphasize the necessity of customized approaches to comprehend and manage DVT in young adults. It is important to focus on factors that may increase the risk of PTS and recurrence.

## Data Availability

The original contributions presented in the study are included in the article/Supplementary Material, further inquiries can be directed to the corresponding authors.

## References

[1] Vrints CJM. Deep venous thrombosis and endothelial dysfunction in cancer: prevention and early initiated rehabilitation should be integral to a cardio-oncology programme. Eur J Prev Cardiol. (2022) 29:1244–7. 10.1093/eurjpc/zwab117 34463767

[2] Mulatu A Melaku T Chelkeba L. Deep venous thrombosis recurrence and its predictors at selected tertiary hospitals in Ethiopia: a prospective cohort study. Clin Appl Thromb Hemost. (2020) 26:1076029620941077. 10.1177/1076029620941077 32931311 PMC7495521

[3] Stein PD Matta F Hughes MJ. In-hospital mortality with deep venous thrombosis. Am J Med. (2017) 130:596–600. 10.1016/j.amjmed.2016.10.030 27894736

[4] Cohen CT Sartain SE Sangi-Haghpeykar H Kukreja KU Desai SB. Clinical characteristics and outcomes of combined thrombolysis and anticoagulation for pediatric and young adult lower extremity and inferior vena cava thrombosis. Pediatr Hematol Oncol. (2021) 38:528–42. 10.1080/08880018.2021.1889729 33646916

[5] Abood KK Paul MR Kuo DJ. Deep vein thrombosis in a young, healthy baseball catcher: a case report and review of the literature. J Pediatr Hematol Oncol. (2019) 41:321–3. 10.1097/MPH.0000000000001113 29401105 PMC7216754

[6] Rosenthal J Kilburn T Jackson W Lune PV Nutting A Pacheco L. Double trouble: a young female with extensive deep venous thrombosis. Am J Med. (2023) 136:e113–4. 10.1016/j.amjmed.2023.01.040 36828208

[7] McLafferty RB. Endovascular management of deep venous thrombosis. Perspect Vasc Surg Endovasc Ther. (2008) 20:87–91. 10.1177/1531003507313304 18388009

[8] Kim KA Choi SY Kim R. Endovascular treatment for lower extremity deep vein thrombosis: an overview. Korean J Radiol. (2021) 22:931–43. 10.3348/kjr.2020.0675 33660456 PMC8154777

[9] Vorob’eva NM Panchenko EP Dobrovol’skiĭ AB Titaeva EV Khasanova ZB Konovalova NV Independent predictors of deep vein thrombosis (results of prospective 18 months study). Kardiologiia. (2010) 50:52–8.. PMID: . 21591393

[10] Raymundo SRO Cabral VS Cavalieri RF Reis Neto F. Thrombolysis for deep venous thrombosis associated with inferior vena cava agenesis in a young patient. BMJ Case Rep. (2019) 12(5):e229840. 10.1136/bcr-2019-229840 31129644 PMC6536156

[11] Kahn SR. The post-thrombotic syndrome. Hematology Am Soc Hematol Educ Program. (2016) 2016:413–8. 10.1182/asheducation-2016.1.413 27913509 PMC6142466

[12] Vedantham S Goldhaber SZ Julian JA Kahn SR Jaff MR Cohen DJ Pharmacomechanical catheter-directed thrombolysis for deep-vein thrombosis. N Engl J Med. (2017) 377:2240–52. 10.1056/NEJMoa1615066 29211671 PMC5763501

[13] Huang CY Hsu HL Kuo TT Lee CY Hsu CP. Percutaneous pharmacomechanical thrombectomy offers lower risk of post-thrombotic syndrome than catheter-directed thrombolysis in patients with acute deep vein thrombosis of the lower limb. Ann Vasc Surg. (2015) 29:995–1002. 10.1016/j.avsg.2015.01.014 25765634

[14] Kuo TT Huang CY Hsu CP Lee CY. Catheter-directed thrombolysis and pharmacomechanical thrombectomy improve midterm outcome in acute iliofemoral deep vein thrombosis. J Chin Med Assoc. (2017) 80:72–9. 10.1016/j.jcma.2016.08.012 28025027

[15] Hager E Yuo T Avgerinos E Naddaf A Jeyabalan G Marone L Anatomic and functional outcomes of pharmacomechanical and catheter-directed thrombolysis of iliofemoral deep venous thrombosis. J Vasc Surg Venous Lymphat Disord. (2014) 2:246–52. 10.1016/j.jvsv.2014.02.003 26993382

[16] Wagenhäuser MU Dimopoulos C Antakyali K Meyer-Janiszewski YK Mulorz J Ibing W Clinical outcomes after direct and indirect surgical venous thrombectomy for inferior vena cava thrombosis. J Vasc Surg Venous Lymphat Disord. (2019) 7:333–43.e2. 10.1016/j.jvsv.2018.11.005 30853561

